# Comparison of Endothelial Progenitor Cells in Parkinson's Disease Patients Treated with Levodopa and Levodopa/COMT Inhibitor

**DOI:** 10.1371/journal.pone.0021536

**Published:** 2011-06-28

**Authors:** Phil Hyu Lee, Han-Soo Kim, Ji E. Lee, Youjeong Choi, Jin Yong Hong, Hyo Suk Nam, Young H. Sohn, Hyun Ok Kim

**Affiliations:** 1 Department of Neurology and Brain Research Institute, Yonsei University College of Medicine, Seoul, Korea; 2 Department of Laboratory Medicine and Yonsei Cell Therapy Center, Yonsei University College of Medicine, Seoul, Korea; 3 Severance Biomedical Science Institute, Yonsei University, Seoul, Korea; University of North Dakota, United States of America

## Abstract

**Background:**

Levodopa treatment in Parkinson's disease (PD) increases in serum homocysteine levels due to its metabolism via catechol O-methyltransferase. Endothelial progenitor cells (EPCs) have the capacity to differentiate into mature endothelial cells and are markers for endothelial functions and cardiovascular risks. Along with traditional vascular risk factors, hyperhomocysteinemia is known to decrease the level of EPCs. In the present study, we hypothesized that that levodopa-induced hyperhomocysteinemia leads to a change in EPC levels.

**Methodology/Principal Findings:**

We prospectively enrolled PD patients who had been prescribed either levodopa/carbidopa (PD-L group, n = 28) or levodopa/carbidopa/COMT inhibitor (PD-LC group, n = 25) for more than 1 year. The number of circulating EPCs was measured by flow cytometry using dual staining of anti-CD34 and anti-KDR antibodies. The EPCs were divided into tertiles based on their distributions and a logistic regression analysis was used to estimate independent predictors of the highest tertile of EPCs. The number of endothelial progenitor cells was significantly decreased in PD-L patients (118±99/mL) compared with either PD-LC patients (269±258/mL, *p* = 0.007) or controls (206±204/mL, *p* = 0.012). The level of homocysteine was significantly increased in PD-L patients (14.9±5.3 µmol/L) compared with either PD-LC patients (11.9±3.0 µmol/L, *p* = 0.028) or controls (11.1±2.5 µmol/L, *p* = 0.012). The level of homocysteine was negatively correlated with endothelial progenitor cell levels (*r* = −0.252, *p* = 0.028) and was an independent predictor of the highest tertile of endothelial progenitor cell levels (OR; 0.749 [95% CI: 0.584–0.961]).

**Conclusions/Significance:**

These data indicate that a higher consumption of EPC for restoration of endothelial damage may be associated with chronic levodopa treatment in PD patients.

## Introduction

Levodopa treatment is the gold standard therapy in patients with Parkinson's disease (PD), with its controlling motor symptoms, improving quality of life, and prolonging patient's life- expectancy. Levodopa therapy, however, causes increase in serum homocysteine level due to its metabolism via catechol O-methyltransferase [1]. Hyperhomocysteinemia causes endothelial dysfunction-mediated vascular impairment and is a risk factor for vascular disease and cognitive impairment in elderly people [2]. Indeed, recent reports demonstrate that hyperhomocysteinemia in PD may be associated with an increased prevalence of coronary artery disease, carotid artery hypertrophy, peripheral neurodegeneration, and increased cerebrovascular resistance [3]–[6].

Endothelial progenitor cells (EPCs) originate in the bone marrow, and appear to have the ability to be mobilized and the capacity to differentiate into mature endothelial cells in response to vascular trauma and ischemic insults [7]. Additionally, it has been suggested that circulating EPCs may be a marker for endothelial function and cardiovascular risk [8]. Along with traditional vascular risk factors, hyperhomocysteinemia is known to decrease the level of EPCs [9]. In this study, we compared the EPC levels of PD patients treated with levodopa and levodopa/COMT inhibitor to test the hypothesis that hyperhomocysteinemia associated with chronic levodopa treatment leads to a change in EPC levels.

## Methods

### Subjects

Fifty-three PD patients and 23 healthy controls were prospectively enrolled from March 2010 to November 2010 at a university hospital. PD was diagnosed according to the UK Brain Bank Criteria [10], and patients had been prescribed either levodopa/carbidopa or levodopa/carbidopa/COMT inhibitor (Stalevo® or levodopa/carbidopa and COMTAN®) for more than 1 year. The PD patients were divided into a levodopa/carbidopa (PD-L)-treatment group or a levodopa/carbidopa/COMT inhibitor (PD-LC)-treatment group. Patients in the PD-LC group were prescribed either Stalevo® or levodopa/carbidopa and Comtan®. Parkinsonian motor symptoms were assessed using the Unified PD Rating Scale Part III (UPDRS-III) while patients were in the ‘on’ state, and the total medication dosages were calculated in levodopa equivalents. All PD patients showed decreased dopamine transporter uptake in the posterior putamen on 18F-FP-CIT PET scans. Healthy elderly volunteers who had no active neurological disorders were recruited by advertisements about the project, or were healthy relatives of patients with movement disorders or dementia.

Data on age and gender, medication history (anti-hypertensive agents, anti-hypoglycemic agents, anti-platelet agents, and statins), smoking history, lipid profiles, body mass index (BMI, kg/m^2^), and high sensitivity C-reactive protein (CRP) were obtained for all participants. Subjects with vascular parkinsonism, parkinsonian-plus syndromes, and PD dementia were excluded. All subjects of PD patients and controls were also excluded with conditions that affect EPCs or homocysteine levels, such as cancer, hepatic or renal insufficiency, symptomatic vascular disease, severe white matter hyperintensities, or multiple lacunes visible on brain MRIs, or if they had been prescribed thiazide, anticonvulsants, or antifolate.

### Ethics statements

The study was approved by the Yonsei University Severance Hospital ethical standards committee on human experimentation. Written informed consent was also obtained from all participants.

### Blood sampling

Blood samples were collected in the morning with fasting state. Plasma homocysteine concentrations were determined by HPLC with fluorescence detection. Serum B12 and folate levels were assessed by immunoassay. After the extraction and purification of DNA, a PCR was performed for the evaluation of 5, 10-methylenetetrahydrofolate reductase (MTHFR) genotype [11].

### Determination of EPC content

**Figure 1:**
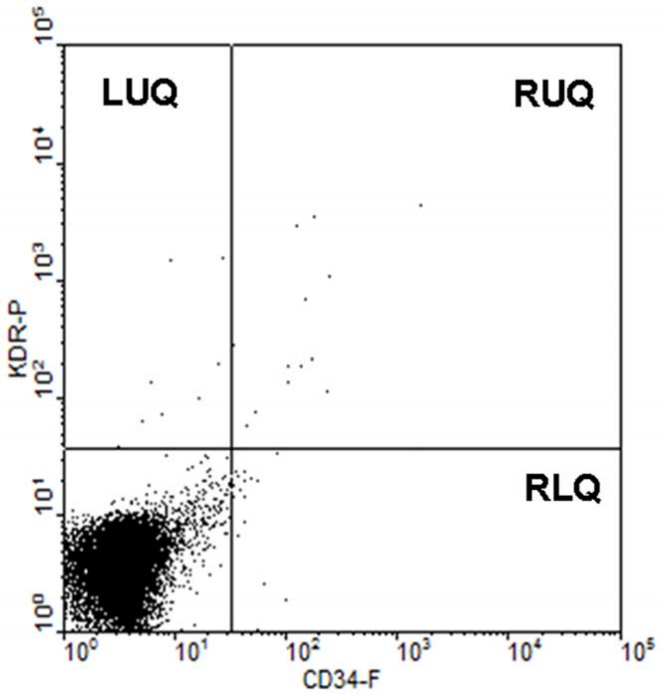
Representative flow cytometric analysis of CD34^+^KDR^+^ cells. KDR-positive cells are displayed in left and right upper quadrants (LUQ and RUQ) and CD34-positve cells are displayed in right upper and lower quadrants (RUQ and RLQ). CD34-and KDR-positive cells are representative in RUQ.

Whole blood (7 ml) was collected in heparin CPT tubes with pre-established density gradient (Vacutainer Cell Preparation Tube, BD, Franklin Lakes, NJ) and processed according to the manufacturer's instructions. After centrifugation at 800× *g* for 20 min at RT, the peripheral blood mononuclear cells (PBMCs) were isolated, washed with PBS, and counted for recovery and viability using Trypan Blue. Since EPCs are characterized by the coexpression of hematopoietic stem cell/progenitor marker (CD34) and endothelial cell lineage markers (KDR) [12], [13], we determined the content of EPC in PBMC by flow cytometry using dual staining with fluorescein-conjugated monoclonal antibodies against CD34/KDR markers (figure 1). Briefly, PBMCs (5 × 10^5^) were incubated with CD34-FITC/KDR-PE (BD) for 20 min at 4°C in a dark room. Nonspecific binding was determined by staining an aliquot of cells with fluorescein-conjugated isotype controls. Following one wash with PBS, cells were fixed with 1% paraformaldehyde and analyzed by CYTOMICS FC-500 (Beckman Coulter, CA). A minimum of 30,000 events was acquired for each analysis in a list mode file format. The data were analyzed using CXP analysis software ver. 2.0 (Beckman Coulter). The absolute counts of EPC were determined by multiplying the frequency of positive cells determined in cytometric analysis by the number of lymphocytes and monocytes as determined by complete blood counts.

### Statistics

The Mann-Whitney or Kruskal-Wallis and Fisher's exact tests were used to compare categorical and continuous variables among groups. Pearson's correlation was conducted to evaluate the relationship between the levels of EPCs and homocysteine. The circulating EPCs were divided into tertiles based on their distributions. A logistic regression analysis in a forward step-wise manner was used to estimate independent predictors of the highest tertile of EPCs. For the mode, subjects with EPC levels in the highest tertile were compared with those in the lower tertile. The independent variables were age, gender, duration of PD and levodopa medication, levodopa dosage, UPDRS III, MMSE, medication use, lipid levels, BMI, genotype, levels of folate and vitamin B12, and CRP. Statistical analyses were performed using commercially available software (SPSS, ver. 18.0), and a p-value of <0.05 was deemed to indicate statistical significance.

## Results

The demographic characteristics of the groups are summarized table 1. No significant differences in age, gender, medication use, smoking, BMI, K-MMSE, and levels of plasma vitamin B6 and B12 were observed. HDL levels were higher in controls than in PD patients. The frequency of the 677C/C and C677C/T genotypes did not differ significantly between the groups, however, 677T/T genotype was significantly less prevalent in control subjects (4.3%) compared with the PD groups (PD-L, 32.1%; PD-LC, 24.0%). CPR was significantly increased in PD-L patients (2.7±4.1) compared with PD-LC patients (1.5±3.1) or controls (1.1±2.0). In patients with PD, no significant differences in medication duration (3.1±3.8 vs. 2.6±2.9), PD duration (4.4±4.8 vs. 3.8±3.1), levodopa equivalent dosage (633.0±230.2 vs. 592.8±120.1), and UPDRS III scores (18.9±8.8 vs. 16.2±5.9) were found between PD-L and PD-LC groups, respectively. However, the daily levodopa dose was higher in the PD-L group (516.1±240.4 vs. 408.0±110.5, *p* = 0.025).

**Table 1:** Demographic characteristics in patients with Parkinson's disease and controls.

	PD-L (n = 28)	PD-LC (n = 25)	Control (n = 23)	*p* *
Age (years)	68.5±5.4	67.2±6.6	66.8±4.9	NS
Gender (female)	18 (64.3%)	12 (48.0%)	18 (78.3%)	NS
Disease duration (years)	4.4±4.8	3.8±3.1	-	NS
UPDRS-III	18.9±8.8	16.2±5.9	-	NS
Duration of levodopa therapy (years)	3.1±3.8	2.6±2.9	-	NS
Levodopa daily dose (mg/day)	516.1±240.4	408.0±110.5	-	0.025
COMT inhibitor daily dose (mg/day)	-	600.0±0.0	-	-
Levodopa equivalent dose	633.0±230.2	592.8±120.1	-	
BMI (kg/m^2^)	24.0±2.7	23.4±3.1	24.7±3.1	NS
Total cholesterol (mg/dL)	189.3±46.2	177.0±37.8	186.7±48.2	NS
Triglyceride (mg/dL)	131.9±53.7	119.2±45.4	106.1±58.1	NS
HDL (mg/dL)	49.2±13.0^a^	44.8±8.1^a^	67.1±33.4	0.007
LDL (mg/dL)	112.9±34.0	108.0±31.3	111.6±28.5	NS
Smoking History	4 (14.3%)	2 (8.3%)	3 (13.0%)	NS
Medication use				
Anti-hypertensive	12 (42.9%)	11 (44.0%)	12 (52.2%)	NS
Anti-hypoglycemic	5 (17.9%)	4 (16.0%)	1 (4.3%)	NS
Statins	4 (14.2%)	7 (28.0%)	7 (30.4%)	NS
MTHFR genotype				
677CC	8 (28.6%)	6 (24.0%)	9 (39.1%)	NS
677CT	11 (39.3%)	13 (52.0%)	13 (56.5%)	NS
677TT	9 (32.1%)	6 (24.0%)	1 (4.3%)	0.036
Vitamin B12 (pg/ml)	669.1±346.1	729.8±283.2	774.4±299.1	NS
Folate (pg/ml)	9.0±4.2	10.1±4.5	10.4±4.5	NS
CRP	2.7±4.1	1.5±3.1	1.1±2.0	0.035
MMSE	26.8±2.6	26.6±3.2	27.0±2.9	NS
Homocystein (µmol/L)	14.9±5.3^b^	11.9±3.0	11.1±2.5	0.012

Values are mean ± SD. PD-L = Parkinson's disease with levodopa treatment; PD-LC = Parkinson's disease with levodopa/COMT inhibitor treatment; BMI = body mass index; HDL = high-density lipoprotein; LDL = low-density lipoprotein; MMSE = Mini-Mental State Examination.

*The Mann-Whitney and Fisher's exact tests were used to compare categorical and continuous variables between groups. The Kruskal-Wallis was used compared continuous variable among group, and the Mann-Whitney test was then performed to compare significant Kruskal-Wallis results:

a From controls at *p*<0.01.

b From cases with PD-LC or controls at *p*<0.05. NS =  not significant.

**Figure 2:**
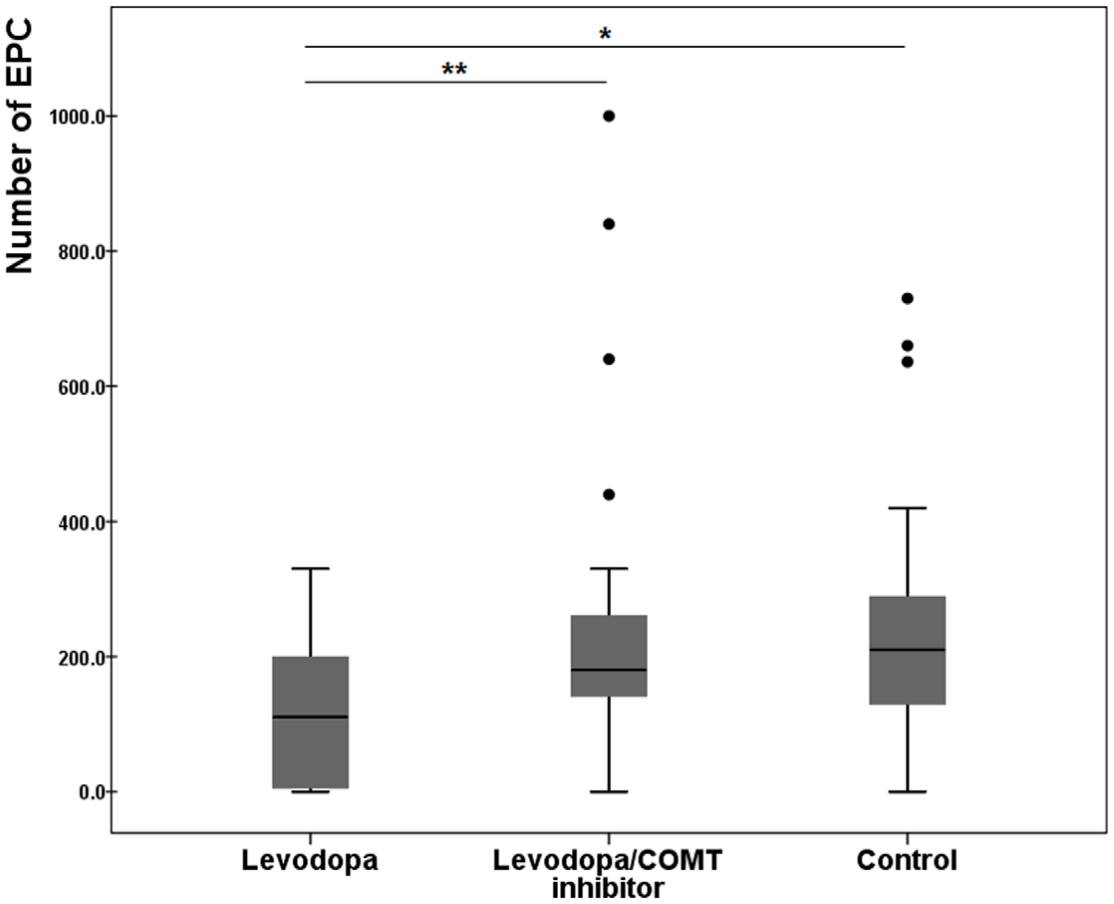
The number of endothelial progenitor cells (EPCs) in patients with PD and controls. Box-and-whisker plot (A) showed that the number of EPC was significantly decreased in PD patients treated with levodopa compared with PD patients treated with levodopa/COMT inhibitor (p<0.01) or control subjects (p<0.05). The black horizontal line in each box represents the median, with the boxes representing the interquartile range and the whiskers represent the maximum. Outliers are recorded individually. *p<0.05, **p<0.01.

**Figure 3:**
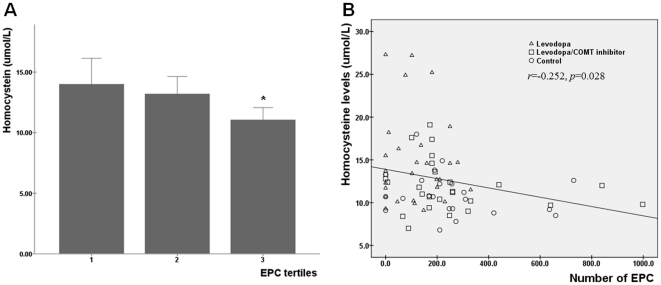
Association between levels of endothelial progenitor cells (EPCs) and homocysteine. Among the EPC tertile subgroups, the homocysteine level was significantly lower in the highest EPC tertile compared with the lowest (11.1 ± 2.5 vs. 14.0 ± 5.5 µmol/L, A). Correlation analysis of the EPC level showed a significantly negative correlation with the level of homocysteine (B). *p<0.05.

The number of EPCs was significantly decreased in PD-L patients (118±99/mL) compared with either PD-LC patients (269±258/mL, *p* = 0.007) or controls (206±204/mL, *p* = 0.012; figure 2). The level of homocysteine was significantly increased in PD-L patients (14.9±5.3 µmol/L) compared with either PD-LC patients (11.9±3.0 µmol/L, *p* = 0.028) or controls (11.1±2.5 µmol/L, *p* = 0.012). Among the EPC tertile subgroups, the homocysteine level was significantly lower in the highest EPC tertile compared with the lowest (11.1 ± 2.5 vs. 14.0 ± 5.5 µmol/L, *p* = 0.027; figure 3A). The correlation analysis showed that the level of EPC had a significant negative correlation with the level of homocysteine (*r* = −0.252, *p* = 0.028; figure 3B). This association remained significant after adjusting for age (*r* = −0.239, *p* = 0.039), as age was partially associated with homocysteine levels (*r* = 0.214, *p* = 0.064). Additionally, the level of homocysteine showed a positive correlation with levodopa dose (*r* = 0.470, *p*<0.001) and a negative correlation with folate (*r* = −0.213, *p* = 0.065) and vitamin B12 levels (*r* = −0.454, *p*<0.001).

Logistic regression analysis revealed that homocysteine (OR; 0.749 [95% CI: 0.584–0.961], *p* = 0.023), 677C/C genotype (OR; 9.157 [95% CI: 1.777–47.202], *p* = 0.008), and vitamin B12 (OR; 1.003 [95% CI: 1.001–1.005], *p* = 0.008) were independent predictors of the highest tertile of EPCs.

## Discussion

This is the first study evaluating EPC levels in PD patients treated chronically with levodopa and levodopa/COMT inhibitor and their association with homocysteine levels. The major findings were (1) EPC levels were significantly decreased in the PD-L group compared with those in the PD-LC group and (2) the level of homocysteine was negatively correlated with EPC levels and was an independent predictor of the highest tertile of EPC levels. These data indicate that a higher consumption of EPC for restoration of endothelial damage may be associated with chronic levodopa treatment in PD patients.

Several factors including smoking, hypertension, hyperhomocysteinemia, hypercholesterolemia, diabetes, and cerebrovascular diseases are known to be associated with low levels of EPCs [9].^7^ Decreased EPC levels may lead to impaired capacity to repair endothelium and thus increase the cardiovascular risks associated with endothelial dysfunctions. In vitro and in vivo studies suggested that hyperhomocysteinemia had detrimental effects on EPCs quantitatively and qualitatively through induction of apoptosis or interference with signaling pathways regulating proliferation, migration, adhesion and vasculogenetic capacity of EPC [14], [15]. Furthermore, clinical studies demonstrated that EPCs had an inverse relationship with homocysteine levels in patients with ischemic stroke or controls [15], [16]. Nevertheless, those studies merely provided an association of EPCs and homocysteine levels that the decrease in EPCs and elevation in homocysteine share a common cause, not showing a direct causation. The present study demonstrated that EPC levels were significantly decreased in PD-LC patients compared with PD-L patients or control subjects, and the homocysteine level was an independent predictor of the highest EPC tertile. In this regard, our study may provide a direct causation between the levels of EPCs and homocysteine since it is well established that in patients with PD, hyperhomocysteinemia is a consequence of levodopa therapy, but not of underlying vasculopathy. Interestingly, the levels of EPCs and homocysteine in PD-LC patients were comparable to those in controls. This may be secondary to homocysteine-lowering effects by COMT inhibitors via reducing the O-methylation as previous studies, in which COMT inhibitor was treated chronically [17], [18]. Along with previous results, our data suggest that an abnormal endothelial repair process is associated with chronic levodopa treatment-related hyperhomocysteinemia in patients with PD. Additionally, increased CRP in PD-L patients compared to controls or PD-LC patients, which is one of indirect markers of endothelial function, may reflect partially abnormality of endothelial repair mechanism in these patients.

Growing evidence suggests that decreased EPC levels are a significant independent predictor of future cerebrovascular events or atherosclerotic disease progression in patients with coronary artery or chronic kidney disease [19], [20]. In this regard, it is speculated that PD patients receiving levodopa chronically may have an increased cerebrovascular burden. However, this association has been controversial because the pathophysiology of cerebrovascular disease in PD is more complex and multifactorial, with both risk and preventive factors; lower prevalence of smoking and decreased glucose, cholesterol, and blood pressure levels by levodopa may give a protective role, whereas immobility, supine hypertension, and levodopa-related hyperhomocysteinemia may increase cerebrovascular risks [21]. Recently, Jickling and colleagues [22] reported that decreased numbers of EPCs are associated with age-related severe white matter hyperintensities, which is one of the comorbid conditions in PD and more prevalent in PD patients compared to controls [21]. Accordingly, a longitudinal study with neuroimaging markers is required to delineate the association of EPC levels and the future cerebrovascular burden in PD patients chronically receiving levodopa.

Some limitations in the present study need to be considered. First, the sample size with a large variation of EPC levels and the effects of outliers may limit the validity of the conclusion. When we performed subanalysis with elimination of outliers of homocysteine and EPC in each group, the statistical difference among groups was still maintained although the statistical power to detect difference in EPC was decreased. Nevertheless, a large-scale study is warranted to draw a solid conclusion. Second, despite the absence of significant differences in demographic characteristics among groups, we could not completely exclude the influence of confounding factors on the level of EPCs in PD patients, such as dietary factors or physical activities associated with PD conditions. Third, together with the general absence of standardized techniques or markers to identify EPCs [23], the flow cytometry technique used in this study has limitations in the measurement of EPC functional properties compared to cell culture methods, in which the PBMCs are cultured for several days in conditions that selectively favor the growth of EPCs. Thus, our results should be interpreted cautiously with knowledge of the limitations of the flow cytometry technique. Finally, although homocysteine may be the best marker of methylation status, measurement of S-adenosylmethionine and S-adenosylhomocysteine levels would provide further information about the mechanism of the homocysteine effect.

In summary, our study demonstrated that the EPC levels were significantly decreased in PD patients with levodopa treatment than in those with levodopa/COMT inhibitors treatment, and the level of homocysteine was an independent predictor of the highest tertile of EPC levels. A future longitudinal study with a larger sample size is needed to delineate the association of EPC levels and cerebrovascular burden in patients with PD chronically receiving levodopa.
